# Does Overfilling Smooth Inflatable Saline-Filled Breast Implants Decrease the Deflation Rate? Experience with 4761 Augmentation Mammaplasty Patients

**DOI:** 10.1007/s00266-021-02198-3

**Published:** 2021-03-12

**Authors:** Ted Eisenberg

**Affiliations:** Nazareth Hospital, 2375 Woodward Street, Suite 102, Philadelphia, PA 19115 USA

**Keywords:** Saline implants, Breast implants, Implant deflation, Breast augmentation, Overfilling saline implants, Deflation rate of implants

## Abstract

**Background:**

Research suggests that overfilling saline inflatable breast implants may decrease their deflation rates. To date, there has been no large-scale study comparing breast implants filled within vs. beyond the manufacturer’s recommended fill volumes.

**Methods:**

A retrospective comparative study was conducted for 4761 women who underwent aesthetic augmentation mammaplasty by the author. Patients were divided into two groups: Group 1 includes 2960 patients who had breast augmentation between 2002 and 2009 with implants filled within the manufacturer’s recommended fill volumes. Group 2 includes 1801 patients who had breast augmentation between 2011 and 2018. Their implants were overfilled with an average volume between 42 and 50 cc, or 10–13%, respectively.

All surgeries were performed with Mentor Style 1600 saline breast implants (smooth, round moderate profile) through an inframammary incision; implants were placed in the subpectoral (dual) plane. Also evaluated was the yearly increase in deflation rates.

**Results:**

Group I had 119 deflations, representing a deflation rate of 4.02%. Group 2 had 33 deflations, providing a deflation rate of 1.83%. The author found a protective effect in overfilling the device (*p* < 0.00001 and *Z*-score of 4.17). Fold failure was the major reason for deflation in both groups.

The implants filled within the manufacturer’s recommended volume had a yearly increase in deflation rate of 0.41%, not 1% as is commonly reported. The overfilled implants did not have a yearly increase in deflation rate.

**Conclusion:**

Overfilling Mentor Style 1600 saline breast implants (smooth, round moderate profile) 10–13% significantly reduced the deflation rate.

**Level of Evidence III:**

This journal requires that authors assign a level of evidence to each article. For a full description of these Evidence-Based Medicine ratings, please refer to the Table of Contents or the online Instructions to Authors www.springer.com/00266.

## Introduction

The goal of this retrospective comparative study was to determine the advantages of overfilling saline breast implants. This was done by comparing the deflation rates of smooth saline breast implants filled within the manufacturer’s recommended volume with those filled beyond.

Saline breast implants were first made available in 1965, three years after silicone gel implants were introduced to the market [1]. During the 1992–2006 FDA moratorium on silicone gel implants for cosmetic breast surgery, saline implants were the prosthesis of choice in the USA and Canada [2, 3].

Deflation is one of the most common complications associated with inflatable saline-filled breast implants. There is a wide disparity in implant deflation data; reported rates vary from 1.3 to 76% for patients who were followed from 1 to 20 years [3–5]. In Mentor’s post-approval study of 1264 patients, the 5-, 7- and 10-year complication rates for deflation were reported at 9.7%, 16.5% and 24.7%, respectively [6].

Studies have suggested that fold (or crease) failure is the most common cause of deflation in saline implants filled under [7] or to the manufacturer’s recommended fill volume [8]; other studies suggest that overfilling implants beyond the manufacturer’s recommended fill volume may reduce the deflation rate [8–11].

This review reports a single surgeon’s experience over 16 years with one style of saline breast implants from a single manufacturer. It is the largest sampling of patient deflation data to date.

## Material and Methods

This study consists of data from 4761 patients who underwent aesthetic augmentation mammaplasty performed by the same surgeon (the author) between 2002 and 2018. Surgeries were performed under general anesthesia in a hospital on an outpatient basis.

Breast augmentation patients received perioperative antibiotics of cephalosporin, or doxycycline if they were penicillin-allergic. All breast implants used were of the same type: Mentor Style 1600 saline breast implants (smooth, round moderate profile). This implant has an anterior diaphragm valve and is constructed from room-temperature vulcanized (RTV) silicone elastomer made of polydimethylsiloxane. Clinically over 16 years, this product did not appear to be any different. The Mentor Product Insert Data sheet makes no mention of any changes to the characteristics of the implant, and no premarket application (PMA) was made to the U.S. Food and Drug Administration (FDA) for any structural changes to this implant since March 2000.

Implants were inserted through an inframammary incision and placed in the subpectoral (dual) plane after the pocket was irrigated with an antibiotic/saline solution containing gentamicin with bacitracin or cefazolin. All implants were inflated after implantation to the desired size with sterile isotonic saline. The same surgical technique and the same style of breast implants were used over the 16 years; there were no variations.

All deflations reported here were first made known by the patient, who reported a significant and almost immediate breast asymmetry and lack of volume. Most times there was complete implant deflation, but on occasion there was a slow leak resulting in a partial implant deflation. These findings were confirmed by physical evaluation at the in-office consultation and again at the time of surgery, and all deflated implants were returned to the manufacturer for analysis. Mammography and ultrasound were not used to confirm deflation.

To conduct the study, the date of the primary breast augmentation was extracted from the medical records of the patients who reported a deflation. A possible limitation of the follow-up would be the inability to evaluate every single deflation due to a patient relocating to another city or choosing another doctor to repair her deflation.

Patients in this study were divided into two groups. Group 1 consists of 2,960 patients who had breast augmentation surgery between 2002 and 2009 with saline implants, all filled *within* the manufacturer’s recommended fill volume. For example, a 375 cc saline breast implant has a 50 cc fill range and can be filled anywhere from 375 ccs (the minimum) to 425 cc (the maximum).

In this 8-year period, there were 119 deflations in 112 patients. The implants ranged from 225 to 625 cc. The deflated implants had been filled as follows: 30% to the minimum end of the recommended fill volume; 40% had partial or midrange fill volumes; and 30% were filled to the maximum of the recommended fill volume.

When this same group of patients was followed for an additional 8 years (2010–2018), another 67 women reported 72 deflations, thus totaling 179 patients with 191 deflations over the 16-year period.

Group 2 consists of 1,801 patients who had breast augmentation surgery between 2011 and 2018 with saline implants, all filled *beyond* the manufacturer’s recommended fill volume. For example, if a patient chose a volume of 425 cc, the author would fill a 325 cc implant to 425 cc, which is 50 cc (or 13%) beyond the manufacturer’s recommended fill volume of 375 cc. In this 8-year period, there were 33 deflations in 31 patients. The implants ranged from 200 to 575 cc. On average, the deflated implants had been overfilled 10% (42 cc); the median was 13% (50 cc). The overfill volumes ranged from 4 to 18% (15–75 cc).

Because of the time elapsed since Group 1’s initial date of surgery, it was possible to include 16 years of information on patient demographics (Table 1). There was a higher percentage of smokers in Group 1 (not overfilled) than in Group 2, and a higher pregnancy rate in Group 2 (overfilled) than in Group 1. To date, there are no scientific studies implying any correlation between smoking and/or pregnancy and the occurrence of deflation. It is likely that these findings are only incidental and not significant.

**Table 1:** Patient demographics: comparison of Group 1 (179 patients) and Group 2 (31 patients)

	Group 1 (16 years)	Group 2 (8 years)
No. of patients	179	31
Age at deflation	22-70 (38.64 avg.)	23-55 (36.3 avg.)
Height	4’11”-6’1” (5.5” avg.)	5’-6’ (5’5 avg.)
Weight	92-102 lb (135 avg.)	96-200 lb (134.2 avg.)
Smoking history		
Yes	63% (*n* = 121)	23% (*n* = 7)
No	37% (*n* = 70)	77% (*n* = 24)
Previous pregnancy		
Yes	33% (*n* = 64)	66% (*n* = 20)
No	67% (*n* = 127)	34% (*n* = 11)
Family hx breast cancer		
Yes	5% (*n* = 10)	10% (*n* = 3)
No	95% (*n* = 181)	90% (*n* = 28)

Table 2 shows the implant replacement choices. The 191 deflations noted in Group 1 represent 179 patients. The 33 deflations noted in Group 2 represent 31 patients.

**Table 2:** Characteristics of deflation and patient preference for breast implant replacement

	Group 1 (16 years)	Group 2 (8 years)
Number of deflations	191	33
Side of deflation		
Left	53% (*n* =101)	42% (*n* =14)
Right	47% (*n* = 90)	58% (*n* =19)
Replacement tendency		
Both	89% (*n* =170)	70% (*n* = 23)
Only deflated	11% (*n* = 21)	30% (*n* = 10)
Desired change in size		
Same	57% (*n* =109)	49% (*n* =16)
Smaller	9% (*n* =17)	9% (*n* = 3)
Bigger	34% (*n* =65)	42% (*n* =14)
Breast width diameter	17.76 cm avg.	16.4 cm avg.
Size of deflated implant	350–500 cc (432 avg.)	225–700 cc (414 avg.)
Size of implant with fold failure	432 cc avg.	373 cc avg.

## Results

To make an accurate comparison of deflation rates of Groups 1 and 2, only the patients who experienced deflation during the first 8 years of both groups were evaluated. When Group 1 was followed for 8 years (2002–2009), there were 112 patients with 119 deflations. When Group 2 was followed for 8 years (2011–2018), there were 31 patients with 33 deflations.

At the end of 8 years, Group 1 (*n* = 2960) had 119 deflations, representing a rate of 4.02%. Group 2 (*n* = 1801) had 33 deflations, representing a rate of 1.83%. In this latter series, the author found a protective effect in overfilling the device (*p* < 0.00001) and *Z*-score of 4.17. By comparison with Mentor’s study (*n* = 1264), there was a deflation rate of 16.5% at 7 years.

The deflation rates between groups 1 and 2 were not statistically significant at 3 years (*p* < 0.14156; *Z*-score of 1.47). However, deflation rates became significant at 5 years (*p* < 0.01828; *Z*-score of 2.36) and at 7 years (*p* < 0.00038; *Z*-score of 3.56) (Table 3).

**Table 3:** Comparative deflation rates at 3, 5, 7, 8, 10 and 16 years

	3 years	5 years	7 years	8 years	10 years	16 years
Group 1^a^	2.76% (*n* = 1198)	3.52% (*n* = 1991)	3.9% (*n* = 2666)	4.02% (*n* = 2960)	5.1% (*n* = 2960)	6.45% (*n* = 2960)
Group 2^b^	1.74% (*n* = 805)	2.09% (*n* = 1292)	1.95% (*n* = 1641)	1.83% (*n* = 1801)	N/A	N/A
Mentor^a,c^	3.3% (*n* = 1264)	9.7% (*n* = 1264)	16.5% (*n* = 1158)	N/A	24.7% (*n* = 1097)	N/A

^a^Filled within the manufacturer’s recommended fill volume

^b^Filled beyond the manufacturer’s recommended fill volume

^c^Cumulative first occurrence Kaplan–Meier adverse event risk rates in augmentation patients from questionnaires sent yearly to patients

In Table 3, to calculate the percent deflation rate per year for each group, the total number of deflations was divided by the total number of patients who had breast augmentation up to and including that year.

In Group 1 (implants not overfilled), there were 119 deflations over 8 years and 191 over 16 years. The deflation rate steadily increased on average 0.41% per year. In Group 2 (implants overfilled 10–13%), there were 33 deflations. The deflation rate on average did not significantly increase per year. Of interest, in Mentor’s study the deflation rate steadily increased on average of 2.42% per year.

**Fig. 1:**
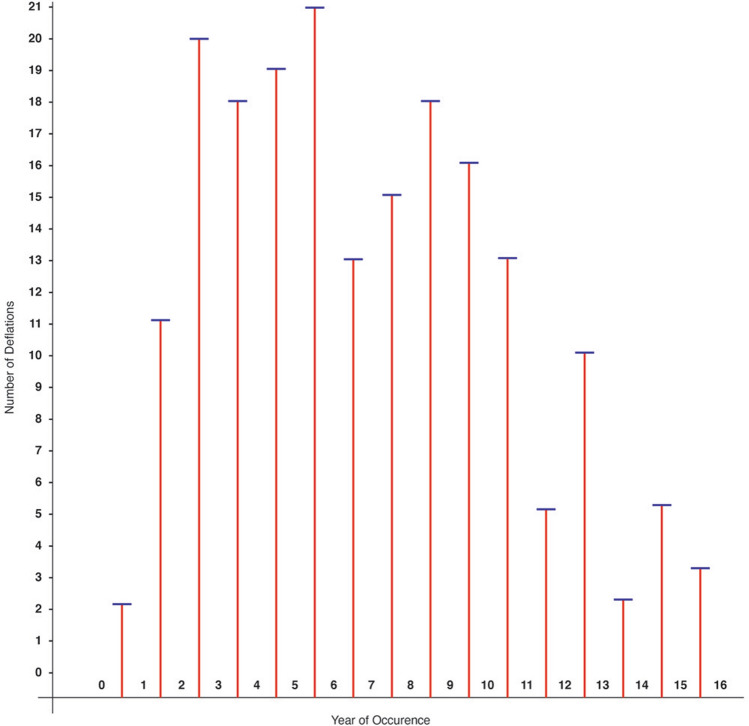
In Group 1 (*n* = 2960, 191 deflations) not overfilled, deflation data are reported for the entire 16-year follow-up. The deflation rate steadily increased on average 0.41% per year

**Fig. 2:**
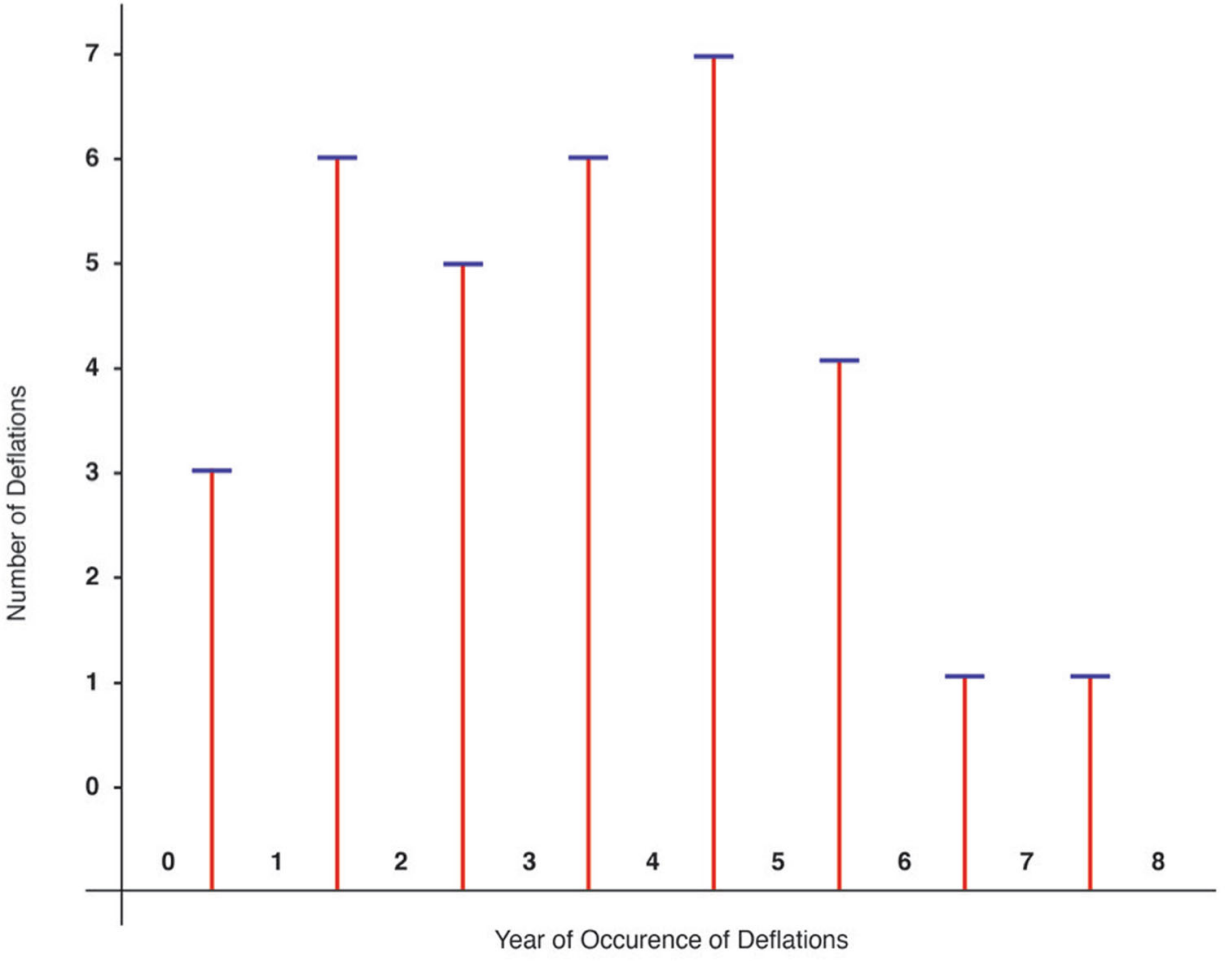
In Group 2 (*n* = 1801, 33 deflations) overfilled 10–13%, the deflation rate on average did not significantly increase over the 8 years

The number of deflations and their year of occurrence are documented in Figs. 1 and 2. In Group 1, for example, 11 deflations occurred between the first and second years (Fig. 1). In Group 2, for example, there were 6 deflations that occurred between the first and second years (Fig. 2).

Over the corresponding 8-year periods, in Group 1 deflation occurred on average at 4.47 years; the range of deflation was 3 days to 8 years. On average in Group 2, deflation occurred at 3.4 years, and the range of deflation was 0.25–7.33 years.

Fold failure accounted for most of the deflations in Group 1 and Group 2. Other categories included undetermined causes, a tear or defect adjacent to the valve, spontaneous rupture of the implant, a tear or defect around the vulcanized silicone dots which hold the leaflet in place over the valve, and iatrogenic causes. See Table 4.

**Table 4:** Causes of breast implant deflation: fold failure was the main cause in both Groups 1 and 2

	Group 1 (*n*=191)^a^	Group 2 (*n*=33)^b^
Fold failure	55.5% (*n*=106)	39.4% (*n*=13)
Adjacent valve tear	15.7% (*n*=30)	21.2% (*n*=7)
Leaflet tear	2.1% (*n*=4)	15.2% (*n*=5)
Spontaneous rupture	4.7% (*n*=9)	12.1% (*n*=4)
Undetermined	21.5% (*n*=41)	12.1% (*n*=4)
Iatrogenic	0.5% (*n*=1)	None

^a^Implants filled within manufacturer’s recommendation; patients followed for 16 years

^b^Implants filled beyond manufacturer’s recommendation; patients followed for 8 years

Although all deflated implants were sent to Mentor for analysis, the company did not consistently provide a report stating the cause of the event. While the causes of deflation noted here are largely based upon clinical observation, when reports were received they were consistent with the author’s findings.

## Discussion

Since the return of silicone breast implants to the US and Canadian markets in 2006, the author has continued to offer his patients the option of saline breast implants. Many women chose this option for the following reasons. Patients appreciated that they would know if their saline implant was deflated without the need for an MRI or ultrasound. Women also liked the smaller incision required for saline implants, because they are inflated after implantation. The lower cost was another incentive. Patients, based on a postoperative survey, were generally satisfied with their aesthetic result. Technically, the author appreciated the ability to make small volume adjustments in patients with minimal asymmetry.

Deflation of saline breast implants is one of the most common complications and biggest concerns for patients and surgeons. Reported deflation rates and the approaches to breast augmentation vary widely. Reported variables include implant style, type and manufacturer; operative technique; incision site; implant placement; and reason for surgery—whether cosmetic or reconstructive [3].

Mladik suggests that the ideal study of deflation rates would have minimal variables and 100% follow-up. In the meantime, plastic surgeons must continue to depend upon their clinical experience [12].

In this study, the variables were largely controlled. In every single case, Mentor Style 1600 saline breast implants (smooth, round moderate profile) were used for cosmetic breast augmentation. The operative technique was consistent through every surgery and performed by the same surgeon; all implants were placed in a dual plane through an inframammary incision.

There was some variability in follow-up in both Groups 1 and 2. Most of the patients (85%) had a manufacturer’s warranty which helped to defray the cost of replacing a deflated implant, a fact that would theoretically incentivize their return to the original surgeon. A possible limitation of the follow-up would be the inability to evaluate every single deflation due to a patient relocating to another city or choosing another doctor to repair her deflation.

### Fill Volumes

In Group 1, the 191 deflated saline implants, which represent 179 patients followed for 16 years, ranged in size from 225 to 625 cc; 30% had been filled to the minimum, 30% to the maximum, and 40% in between the manufacturer’s recommended range. There was no significant difference in the deflation rates of breast implants filled to the manufacturer’s lowest recommended volume compared to those filled to the highest recommended volume, but it was noteworthy that 70% were filled below the maximum range.

In the 1980s, manufacturers developed their fill recommendations for saline implants by adding 25–50 cc to the volume they had previously set for the same size silicone gel implant. Al-Sabounchi suggested that this volume did not reflect the volume needed to adequately fill out the implants in vivo [3].

Lavine and others found that underfilling saline implants contributed to stress points along the wrinkles and eventual deflation [13]. Since 1991, to avoid the formation of folds (the most common cause of deflation), manufacturers have therefore recommended not to underfill inflatable implants [14].

A 2006 in vitro study found that when saline implants were overfilled beyond the manufacturer’s recommendation, there were no significant differences in the mechanical properties of the implant shell (strength, elasticity and toughness). This study also suggested that doing the same study in vivo would determine the effect of overfilling on implant shells [8].

Other studies examined the impact of overfilling saline implants on their longevity and failure rate and suggested that to obtain optimal volume (less wrinkling) most implants required overfilling [10, 11, 13, 15, 16].

In Lavine’s study of 917 patients with Mentor Style 1600 implants, almost all implants were routinely overfilled by at least 25 cc and usually by 50–75 cc to prevent postoperative in situ wrinkling of the implant. His overall deflation rate was 0.56% [13]. Stevens et al. reported an average overfill of 5–15% [17]. Lantieri found a statistically significant positive difference when implants were overfilled in ranges up to 20% compared to not overfilled [7].

In this study, the average overfill of the implants in Group 2 was 10% (42 cc) and the median overfill was 13% (50 cc). By clinical observation, this overfill volume seemed to consistently produce less wrinkling and folding of the implant. In vivo, the implants also looked and felt smoother. There was no evidence of scalloping, which can occur when the implants are excessively overfilled.

### Implant Deflation Rate and Implant Longevity

In this comparative study, the implants that were not overfilled (Group 1) had a deflation rate at 8 years of 4.02%, and those that were overfilled (Group 2) demonstrated the protective effect and had a statistically significant deflation rate at 8 years of 1.83% (*p* < 0.00001).

The author’s results were statistically significant compared to Mentor’s study, even when breast implants (Group 1) were filled *within* the recommended fill volume. (Refer to Table 3.) For example, at 10 years the deflation rate for Group 1 was 5.1% compared to Mentor’s rate of 24.7% (*p* < 0.00001) and a *Z*-score of  − 18.17.

It may be possible that the author’s deflation rates were markedly lower than those of Mentor, even when filled within the recommended fill volume, because while the author’s study reflects the same surgeon doing the same technique with the same product Mentor’s was a “multicenter” clinical study with unknown variables.

It is not known if the surgeons involved in Mentor’s multicenter study have performed any surgical practices that might compromise the product integrity of saline implants, as described in Mentor’s June 2019 Product Insert Data Sheet. These contraindicated practices include placing more than one implant in the breast pocket (stacking), making injections into the implant, altering the implant shell or valve; placing drugs or substances inside the implant other than sterile saline for injection; and allowing the implant to come in contact with Betadine Antiseptic (Purdue Frederick Co.). Other warnings by Mentor include performing closed capsulotomy, which could result in implant damage, deflation and folds; reusing or re-sterilizing; attempting to repair a damaged prosthesis; contacting the implant with a disposable, capacitor-type cautery device; underfilling the prosthetic, which could enhance fold failure with subsequent deflation; and using endoscopic placement or a periumbilical approach in placement of the implant [6].

The author follows these guidelines and has never done any of these contraindicated practices.

There are minimal data quantifying the increase in yearly deflation rates. One study suggests that the deflation rate increases 1% a year—equal to 10% at 10 years [18]. In the Mentor post-approval study (implants not overfilled), the average increase in deflation rate was 2.42% per year over 10 years.

To calculate the average increase in deflation rate per year for each group in this study, the deflation rate percentage was divided by the total number of years studied. In Group 1 (not overfilled), the average deflation rate increase was 0.41% per year when viewed over 16 years. In Group 2 (implants overfilled), there was no significant increase in the yearly deflation rate from the second year on, and the rate stayed fairly even at 0.23% over 8 years. (Refer to Table 3.)

In Group 1 (not overfilled implants) from years 3 to 6, there was an average of 20 deflations per year. From years 7 to 11, deflations dropped to 12 per year. From years 12 to 16, there was a sharp decline to an average of 5 per year. (Refer to Fig. 1.)

In Group 2 (overfilled implants), from years 1 to 6, there was an average of 5 deflations per year. In the last two years studied, there was a sharp decline to 1 per year. (Refer to Fig. 2.)

Regarding implant longevity, while two studies before 2000 reported that overfilling was not a protective measure against deflation [2, 19], Al-Sabounchi, through Kaplan–Meier survival analysis, found a statistically significant increase in the longevity of implants when they were overfilled or filled to the manufacturer’s maximum recommended volume [3].

**Fig. 3:**
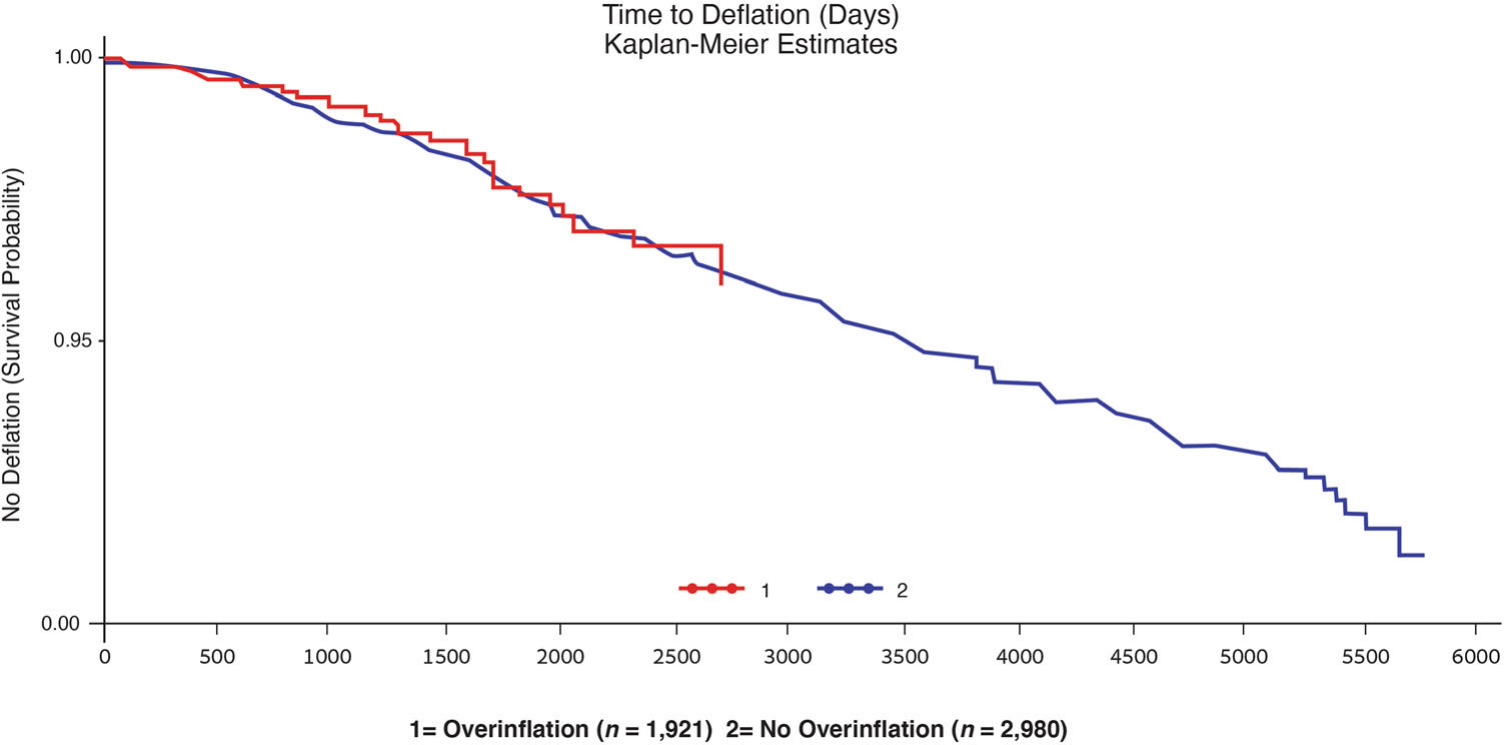
Kaplan–Meier estimate of time to deflation (days). There was no significant increase in longevity (*p* = 0.8966) between implants that were filled within versus beyond the recommended volumes

Although there was not an increase in the deflation rate per year in the overfilled implants in this study, Kaplan–Meier evaluation of Groups 1 and 2 could not discern a statistically significant increase in implant longevity. There is no difference in the time to deflation between patients with and without overinflation (*p* = 0.8966). The duration of follow-up is substantively longer in the no-overinflation group (5,840 days) compared to the overinflation group (2,920 days), which is why the time-to-event graph has only one curve at the end. The time to deflation in the 33 and 191 deflations in the 8-year overinflated and 16-year not overinflated groups, respectively, was linear (Fig. 3).

### Causes of Deflation

For Groups 1 and 2, the principal cause of saline breast implant deflation was fold failure: 55.5% (*n* = 106) in Group 1 and 39.4% (*n* = 13) in Group 2. A fold in the implant shell around the perimeter can cause cyclic fatigue and implant rupture. This cause of leakage has been described in the literature since 1980 and cited as the most common cause of deflation [20].

Other causes of deflation included a tear or defect adjacent to the fill valve and a tear or defect around the hard vulcanized silicone droplet that holds the leaflet in place over the valve. These three causes account for 73% of Group 1's and 76% of Group 2's deflations. The 12–21% of the deflations categorized as “undetermined” were believed by the author to be fold failures not obvious to the naked eye. Overfilling saline breasts implants might allow for slightly less movement at these points, reducing the incidence of cyclic fatigue and deflation. (Refer to Table 4.)

All implants in this study were submerged preoperatively in an antibiotic/saline solution prior to placement to test the competency of the fill valve. Of the 224 deflations reported in Groups 1 and 2, 37 were caused by a leak adjacent to the valve, but none from an incompetent valve.

Another reported cause of deflation included spontaneous rupture from a rent in the side of the implant. There was one iatrogenic deflation caused by wound closure in Group 1. Iatrogenic injuries associated with implant deflation include closed capsulotomy [19], needle damage to the implants during wound closure [21], breast biopsy and mammography.

In the literature, time and trauma (from high impact injuries like auto accidents or falls) have also been reported as causes of implant rupture [2, 16]. Greenwald suggested that time contributes to changes in silicone shell strength, leading to progressive weakening of the shell [8]. Except for this one study, every article reviewed for this paper attributed deflations to mechanical factors and not to time.

While Worseg [14] and Gutwoski [19] observed that submammary placement compared with submuscular placement was not a risk factor for deflation. Mladick found a significant difference in the deflation rates for implants placed subglandularly (4%) vs. subpectorally (1.8 %) [12]. All patients in this study had saline implants placed in the dual plane (partially subpectoral, partially subglandular).

Although Lantieri suggested that capsular contraction may contribute to rupture and deflation, he stated that there has never been any correlation demonstrated between the deflation rate and the capsular contracture rate [7].

Of interest, Worseg had 10 patients who were able to specify their speed of deflation as sudden, while 6 noticed a gradual loss of breast volume over time [14]. Although the speed of deflation was not evaluated in the study reported here, anecdotally it was observed that for most patients the speed of deflation was rapid. On a rare occasion, the rate was slower and seemed to be more associated with those patients who had a deflation at the vulcanized silicone dot by the leaflet.

### Implant Look and Feel

A major concern for patients is that they not have visible or palpable rippling of the implant under the skin. Subjectively, overfilling saline implants often seemed to minimize this rippling, which would be consistent with the studies of Young [16] and Codner [22].

Overfilling can create scalloping around the periphery of the implant, demonstrating the need for an optimal fill volume [23]. Clinical judgment at the time of implantation is needed to limit excess hardness due to overfill.

The author’s clinical observation in vivo shows that 10–13% overfill seems to be the “sweet spot” of fill for this type of implant. It produces less rippling under the skin and no scalloping or hardness of the implant. When a patient chooses a volume of 425 cc, for example, the author fills a 325 cc implant to 425 cc, which is 50 cc (or 13%) beyond the manufacturer’s recommended fill volume of 375 cc.

Through outcome surveys and ultrasound evaluation, Swanson objectively found that the visible and palpable rippling rates of silicone gel and saline implants were similar [24]. In his study, the saline implants were filled *to* the manufacturer’s recommended fill volume. It would be useful to have a similar study comparing *overfilled* saline implants with silicone gel implants to determine if overfilled saline implants demonstrate less visible and palpable rippling rates than silicone.

## Conclusion

Overfilling Mentor Style 1600 saline breast implants (smooth, round moderate profile) an average of 10–13% significantly reduced the deflation rate. Overfilling may also have the advantage of reducing visible and palpable rippling. Further studies would be necessary to determine if overfilling produces the same benefit in other saline inflatable breast implants on the market.
